# Hyperglycemia Modulates the Expression of MAPK13, TSP1, and CXCR2 During Wound Healing in Sprague Dawley Rats

**DOI:** 10.3390/biology15010026

**Published:** 2025-12-23

**Authors:** Tommy Tran, Jaylan Patel, Vy Ho, Betelhem Teshome, Vikrant Rai

**Affiliations:** 1 College of Osteopathic Medicine of the Pacific, Western University of Health Sciences, Pomona, CA 91766, USA; tommy.tran@westernu.edu (T.T.); jaylan.patel@westernu.edu (J.P.); vy.ho@westernu.edu (V.H.); 2 Department of Translational Research, Western University of Health Sciences, Pomona, CA 91766, USA; bteshome@westernu.edu

**Keywords:** hyperglycemia, diabetic foot ulcer, MAPK13, TSP1, CXCR2

## Abstract

Diabetic foot ulcers (DFUs) are a major complication of diabetes, affecting 19–34% of diabetic patients in their lifetime. Decreased angiogenesis, altered extracellular matrix (ECM) remodeling, and chronic inflammation underlie the pathophysiology of nonhealing DFU. Further, MAPK13, which regulates matrix remodeling and promotes inflammation, CXCR2, an IL-8 receptor that drives inflammation, and thrombospondin-1 (TSP-1), which inhibits angiogenesis, play a role in wound healing, but the effect of hyperglycemia on the expression of these mediators during wound healing is not well understood. This study aims to investigate the effects of hyperglycemia on the expression of MAPK13, CXCR2, and TSP-1. Type 2 diabetes was induced in high-fat-fed Sprague Dawley rats using low-dose streptozotocin. Cutaneous wounds were created on the dorsum of control and diabetic rats. Tissue samples were collected during and after wound healing (*n* = 7 per group; total = 28) and used in this study to investigate the gene and protein expression of MAPK13, CXCR2, and TSP-1 in normal and healed skin using RT-qPCR and immunostaining. The results suggest that hyperglycemia increases the expression of TSP1 and CXCR2, and the effects on MAPK13 are context-dependent. Our results provide potential insights into impaired healing in diabetic wounds and highlight therapeutic targets for chronic DFUs by targeting angiogenesis, ECM remodeling, and chronic inflammation.

## Simple Summary

Diabetes affects millions of people in the United States and can lead to serious complications, including diabetic foot ulcers (DFUs). Diabetic patients experience foot nerve damage that leads to unrecognized trauma, chronic inflammation, and reduced blood flow, resulting in nonhealing wounds and DFU formation. There are many biological pathways that contribute to wound healing and inflammation, but they are not fully understood. This study investigated three factors with critical roles in inflammation and wound healing: MAPK13, a signaling molecule for inflammation; TSP1, a protein preventing new blood vessel growth; and CXCR2, a receptor that recruits immune cells. The results revealed an increased MAPK13, TSP1, and CXCR2 with hyperglycemia. These findings suggest that hyperglycemia modulating expression of these factors contributing to chronic inflammation and decreased angiogenesis may contribute to delayed DFU healing and may be potential therapeutic targets.

## 1. Introduction

Diabetes mellitus is one of the most prevalent diseases in the United States, affecting over 38.4 million Americans in 2021 [1]. Diabetic foot ulcers (DFUs) are a severe complication of diabetes, affecting 19–34% of diabetic patients in their lifetime and causing a serious burden on diabetic patients, including increased morbidity, infection, amputation, and overall decreased quality of life [2]. Diabetes causes peripheral neuropathy, leading to loss of protective sensation in the feet, and results in unrecognized trauma and repetitive stress. This repetitive stress can contribute to foot injury and subsequent DFU formation [3]. Diabetes is also associated with peripheral vascular disease, reducing blood flow and impairing oxygen and nutrient delivery necessary for wound healing [4]. This exacerbates tissue damage and delays healing, resulting in the formation of chronic nonhealing DFUs [5]. Chronic inflammation and impaired immune response contribute to DFU formation, characterized by a prolonged inflammatory phase in wound healing, dysregulated macrophage activity, and increased levels of proinflammatory cytokines [6]. Together, the combination of peripheral neuropathy, peripheral vascular disease, and chronic inflammation leads to the development of DFUs [7]. Although numerous mediators, including proinflammatory cytokines, fibroblast heterogeneity and plasticity, and regulators of angiogenesis, have been implicated in chronic nonhealing DFUs, the interactions among these factors are not completely understood [8,9,10,11].

Physiological wound healing involves four overlapping phases—hemostasis, inflammation, proliferation, and remodeling—orchestrated by various cells like platelets, neutrophils, macrophages, fibroblasts, and keratinocytes to stop bleeding, clear debris, rebuild tissue, and strengthen the scar, ensuring proper repair from initial injury to mature scarring. Platelets adhere to the injury, form a plug, and release growth factors (PDGF, TGF-β). Clotting factors create a fibrin mesh to stabilize the clot. Neutrophils are first responders, engulfing bacteria and dead cells (phagocytosis); macrophages arrive later, clearing neutrophils and debris, and releasing growth factors to signal proliferation; and mast cells release histamine, increasing vessel permeability during inflammation phase. Fibroblasts deposit collagen and extracellular matrix, forming granulation tissue; endothelial cells form new blood vessels; keratinocytes migrate across the wound surface (epithelialization) to form new skin; and myofibroblasts begin to contract the wound during proliferation phase. Fibroblasts/myofibroblasts balance collagen production and breakdown, increasing tensile strength during remodeling phase and then macrophages, fibroblasts, and blood vessels decrease as the wound matures [12,13,14,15].

Mitogen-activated protein kinases (MAPKs) are a family of serine/threonine kinases that regulate cellular responses to many extracellular stimuli, including stress, cytokines, and growth factors [16]. Activation of MAPK signaling influences key processes such as cell proliferation, differentiation, apoptosis, and inflammation [17]. The MAPK signaling pathway consists of three major subfamilies: extracellular signal-regulated kinases (ERKs), c-Jun N-terminal kinases (JNKs), and p38 MAPKs, which mediate signal transduction and cellular response regulation [18]. The p38 MAPK family is associated with inflammation and includes four isoforms: p38α, p38β, p38γ, and p38δ. Activation of MAPK involves a signaling cascade that begins with MAPK kinase kinase (MAP3K) phosphorylating a MAPK kinase (MAP2K), which activates the MAPK to facilitate downstream effects [19]. Among the p38 MAPK isoforms, p38α is the most extensively studied [20]. In macrophages and myeloid cells, p38α signaling is required for IL-10 synthesis, which acts as a negative feedback regulator to suppress inflammation and prevent tissue damage [21,22]. However, MAPK13, also known as p38δ, has emerged as an important modulator of inflammatory signaling and stress responses [23]. Under normal conditions, MAPK13 is activated by environmental stressors and proinflammatory cytokines, leading to phosphorylation and inflammatory downstream effects [24]. However, in diabetic conditions, the MAPK/ERK pathway exhibits dysregulation, amplifying inflammatory gene expression, impairing endothelial function, and disrupting normal angiogenesis, leading to DFU formation [25]. In vascular endothelial cells, chronic hyperglycemia increases reactive oxygen species, in turn activating protein kinase C, which results in pathological activation of p38 MAPK and enhances downstream inflammatory and fibrotic gene expression [26]. MAPK13 is also involved in inflammation as an essential regulator of Toll-like receptor 4-induced cytokine production, increasing inflammation by controlling activation of the extracellular signal-regulated kinase (ERK) 1/2 pathways [27]. Regarding therapeutics, p38α is sensitive to the most common p38 inhibitors, but the p38γ and p38δ isoforms are not affected [20]. The current literature remains unclear about the precise role of MAPK13 in DFU nonhealing and targeted therapeutics.

As mentioned above, decreased angiogenesis is a major contributor to nonhealing DFUs. TSP-1 is a potent antiangiogenic agent that inhibits endothelial migration and proliferation and induces endothelial apoptosis [28]. Increased TSP-1 also enhances inflammation by activating transforming growth factor-β (TGF-β), which in turn promotes arterial stiffening and inhibits angiogenesis [29]. TSP-1 also inhibits angiogenesis through interaction with CD47, leading to reduced endothelial cell proliferation, migration, and tube formation, which are essential for neovascularization and tissue repair [30]. Under normal conditions, its levels are transiently upregulated, but in hyperglycemic conditions, TSP-1 is abnormally upregulated, which can exacerbate DFU formation [31]. Furthermore, in diabetic conditions, hyperglycemia enhances MAPK13 activation, which increases TSP1 expression, further amplifying anti-angiogenic and profibrotic signaling that impairs wound healing and promotes DFU formation [32]. TSP1 is also important because it regulates cell behavior and tissue structure by interacting with extracellular matrix (ECM) proteins, cell surface receptors, and proteases. It can influence cell adhesion, migration, and growth factor activity, and plays a significant role in tissue repair, wound healing, fibrosis, and diseases like cancer and glaucoma. TSP1 achieves this by directly interacting with matrix components like collagens and modulating the activity of enzymes that break down and rebuild the ECM [33].

Interleukin-8 (IL-8) also contributes to chronic inflammation and DFU formation [8]. IL-8 is a potent chemokine and binds to CXCR2, a G-protein-coupled receptor, to induce downstream signaling via G proteins [34]. IL-8 and its receptor, C-X-C chemokine receptor type 2 (CXCR2), play a central role in inflammation by mediating neutrophil recruitment and activation at sites of tissue injury or infection [35,36]. However, there is an increased production of IL-8 in hyperglycemic conditions, activating CXCR2 and prolonging neutrophil activation in wounds [37]. These neutrophil extracellular traps (NETs) in chronic hyperglycemic conditions promote persistent inflammation, excessive release of proteases and reactive oxygen species, and delay resolution of the inflammatory phase, impairing the normal tissue repair process [38]. In DFUs, interleukin-8 (IL-8) has been found to play a significant role in causing chronic inflammation, including activation of the MAPK13 pathway [10], which may suggest a relationship between the two inflammatory activators and warrants further exploration.

Chronic inflammation, attenuated angiogenesis, and altered ECM remodeling are major contributors to DFU formation and impaired wound healing. Understanding the interactions among their mediators, including MAPK13, TSP1, and CXCL8, is crucial. This study aims to characterize the cutaneous tissues from the rat model of diabetes for the expression of MAPK13, CXCR2, and TSP-1, comparing the expression between control and healed tissues from nondiabetic and diabetic rats. We hypothesized that hyperglycemia increases the expression of these mediators, which may contribute to the delayed or nonhealing of DFUs.

## 2. Materials and Methods

### 2.1. Animals and Tissue Processing

This study utilized tissues already collected from ongoing studies in the lab (Protocol# R24IACUC013). The tissues were predetermined for the analysis of inflammation, angiogenesis, and immune response between two groups. Tissues were collected from male and female Sprague Dawley rats obtained from Charles River Laboratories (Wilmington, MA, USA), aged 6–8 weeks, and weighing approximately 180 g. The rats were housed in the Animal Resource Facility at Western University of Health Sciences, Pomona, CA, under a temperature of 22 °C and a 12 h light/dark cycle. Tissues were collected from nondiabetic (ND) control (*n* = 7, control and healed each) and diabetic (D) (*n* = 7, control and healed each) rats with a total of 28 samples. The rats were divided into control and diabetic groups randomly, and the grouping was known to the principal investigator only; others were blinded to the groupings. Based on the power analysis using G*power3.0.10 software with an α value of 0.05, effect size of 0.5, 95% confidence interval, the sample size necessary to have at least 90% power (1-β) to detect a significant change is 7 in each group. The tissues from ND rats were collected during wounding (D0) and then after healing (D14) from the same point (dorsum) and from diabetic rats on D0 during wounding and D21 after healing. Type 2 diabetes was induced in the rats using a low dose of streptozotocin (STZ; 25 mg/kg, dissolved in 0.1 M sodium citrate buffer, pH 4.4; Sigma-Aldrich, St. Louis, MO, USA) injected intraperitoneally (IP) after six weeks on a high-fat diet (as discussed in [39]). The control rats were fed a normal diet (ND; 20% protein, 70% carbohydrate, 10% fat; D12450B, Research Diet Inc., New Brunswick, NJ, USA), while the diabetic rats received a high-fat diet (HFD; 35% carbohydrate, 20% protein, 45% fat; 5.7 kcal/g total; D12451, Research Diet Inc., New Brunswick, NJ, USA), with water ad libitum. Cutaneous wounds on the dorsum equidistant from the vertebral column on both sides were created using a punch biopsy (6 mm × 2 mm). Rats with blood glucose levels less than 250 mg/dL (13.9 mM/L) were not included in the study. Tissue samples were collected during wounding and after healing in 10% formalin and processed using the LEICA ASP6025 Tissue Processor (Deer Park, IL, USA). Tissues were then embedded in paraffin and sectioned using the Leica microtome (Deer Park, IL, USA). The sectioned slides were heated at 60 °C for 1 h. The tissues were also collected in RNA later for RNA extraction and kept at 80 °C until used. This study followed the ARRIVE protocol for grouping, and the rats used in this study.

### 2.2. Hematoxylin and Eosin Staining

Tissues were deparaffinized and rehydrated through sequential washes in xylene, descending concentrations of ethanol (100%, 95%, 80%, 70%), and distilled water. Tissues were stained with hematoxylin for 1 min and washed in running water for 5 min. Then the tissues were dipped in Bluing solution for 10 times and stained in eosin for 2 min. After eosin staining, tissues were washed in sequential ethanol solution and xylene. The slides were air-dried and mounted with CytoSeal 60 (23-244257, Fisher Scientific, Hampton, NH, USA) using cover slips. Stained tissues were imaged using a Leica DM6 light microscope (Deer Park, IL, USA) at 5× and 20× resolution with a scale of 100 μm.

### 2.3. Trichrome Staining

Tissue sections were deparaffinized in xylene and rehydrated as in H and E staining. Following a water rinse, samples were fixed in Bouin’s solution for 1 h at 56 °C. Sections were stained with Weigert’s iron hematoxylin for 10 min and Biebrich scarlet-acid fuchsin for 6 min, then washed and differentiated in phosphomolybdic–phosphotungstic acid solution for 10 min. Then slides were transferred to aniline blue for 4–5 min and immersed twice in 1% acetic acid for 2 min each. After another distilled water rinse, tissues were rapidly dehydrated through ethanol and xylene, air-dried, and mounted with CytoSeal 60 using coverslips. Images were acquired using a Leica DM6 light microscope (Deer Park, IL, USA) at 5× and 20× magnification with a scale of 100 μm. Trichrome staining slides were semi-quantitatively analyzed by two independent researchers blindfolded using a scale of 0 to 5, where 0 = minimal blue staining, 1 = very weak blue staining, 2 = weak blue staining, 3 = moderate blue staining, 4 = strong blue staining, and 5 = very strong blue staining. The average of two results were used in the study.

### 2.4. Quantitative Real-Time Polymerase Chain Reaction

Total RNA was extracted using Trizol reagent (Sigma Aldrich, T9424, St. Louis, MO, USA), and RNA concentration was quantified using the Nanodrop 2000 (Thermo Scientific, Waltham, MA, USA). The cDNA was prepared using the iScript cDNA Synthesis Kit (BioRad, #1708891, Irvine, CA, USA). The primer sequences (Table 1) were obtained from Integrated DNA Technologies (Coralville, IA, USA). RT-qPCR was performed using SYBR Green on a CFX96 RT-PCR system (BioRad Laboratories, Hercules, CA, USA) to evaluate the expression of MAPK13, TSP-1, and CXCR2. Each sample well contained 3 μL of cDNA, 5 μL of SYBR Green, 1 μL of forward primer, and 1 μL of reverse primer, and was centrifuged for 2 min before beginning PCR cycles. The PCR cycling conditions were as follows: 95 °C for 5 min for the initial denaturation, followed by 40 cycles of 30 s each at 95 °C, 30 s at 60 °C for annealing, and 30 s at 72 °C for extension. Fold change in mRNA expression relative to controls was analyzed using 2^−ΔΔCt^ after normalization with 18S. Experiments were performed in triplictaes using all 7 samples.

### 2.5. Immunohistochemistry

Immunostaining was performed using the peroxidase–anti-peroxidase method with a horseradish peroxidase–conjugated secondary antibody. Tissues were deparaffinized and rehydrated as mentioned above. Antigen retrieval was performed with 1% citrate buffer (Sigma Aldrich, C9999, St. Louis, MO, USA) for 15 min on a steamer. After cooling and washing slides with 1× phosphate-buffered saline (PBS), tissues were circled using Pap pen. Endogenous peroxidase activity was neutralized by applying 3% hydrogen peroxide to the tissues for 15 min. Tissues were then washed with PBS twice for 5 min each. Nonspecific blocking was performed using the Vectastain kit Elite ABC kit (Vector Labs, Newark, NJ, USA). Tissues were treated with one drop of IgG blocking solution and incubated for 1 h at room temperature. Goat serum was used as the blocking solution for CXCR2 and TSP-1 staining, and horse serum was used for MAPK13 staining. Primary antibodies were diluted in their respective blocking solutions at the following concentrations: CXCR2 polyclonal antibody (bs-1629R) at 1:200, TSP-1 (18304-1-AP) at 1:100, and MAPK13 (OTI12B2) at 1:200. One drop of primary antibody was added to each tissue and incubated overnight at 4 °C. Slides were then washed with 1× PBS twice for 5 min each, and one drop of secondary antibody was added to the tissues and incubated for 1 h at room temperature. Negative controls without primary antibody (but with secondary), only secondary antibody (but with primary), and IgG antibody were run in parallel. Slides were washed with PBS, and one drop of Vectastain Elite ABC horseradish peroxidase complex solution (Vector Labs, Newark, NJ, USA) was added to the tissues and incubated for 30 min. The ABC solution was prepared and incubated for 30 min before use. Slides were then washed with PBS, and tissues were incubated with AEC (3-amino-9-ethylcarbazole) substrate until a brown-red color developed. Sections were washed with water once and stained with hematoxylin for 20–30 s before being rinsed with running tap water for 5 min and mounted with CytoSeal 60. The tissues were imaged with a Leica DM6 microscope at 5× and 20× resolution. For each tissue, three sections were processed, and three to four images were scanned from each tissue.

### 2.6. Cell Culture and In Vitro Studies

Rat fibroblasts were cultured in a T75 culture flask (ThermoFisher, Waltham, MA, USA) until 90% confluence using complete Dulbecco’s Modified Eagle’s Medium (DMEM) with 10% fetal bovine serum and 1% penicillin-streptomycin in a humidified incubator with 5% CO2 at 37 °C. The cells were trypsinized and plated in a 6-well plate and chamber slides. For PCR, 1 × 10^6^ cells were plated in each well of a 6-well plate, and 8 × 10^3^ cells were plated in each chamber of the chamber slide for immunofluorescence. For immunofluorescence, the cells in the chamber slide were washed with sterile PBS after 24 h, fixed with 10% Formaldehyde, and incubated in 0.01% Triton for 10 min. This was followed by washing with PBS and incubation with blocking solution for 1 h. After tipping off the blocking solution, cells were incubated with MAPK13, TSP1, and CXCR2 overnight at 4 °C, followed by washing with PBS and incubation with Alexa Fluor 594 (ThermoFisher, Waltham, MA, USA) for 30 min. The cells were washed, counterstained with DAPI, and scanned using a Leica Fluorescence microscope. Negative controls (only primary and only Alexa Fluor, separately) were run in parallel. The cells in a 6-well plate were cultured overnight, then treated with hyperglycemic medium (DMEM with 9.0 g/L glucose). The cells for RNA extraction were collected at 24 h and 48 h. The cells in chamber slides were treated with hyperglycemic medium for 24 h. Total RNA was extracted using TRIZOL, cDNA was prepared, and RT-qPCR was conducted as described above. 48 h were chosen to investigate the long term (chronic) effects of hyperglycemia on gene expression while 24 h were chosen for short term (acute) effects.

### 2.7. Statistical Analysis

All data are presented as mean ± SD. The analysis was Performed with one-way ANOVA with a Bonferroni correction using GraphPad Prism 10. The comparison between the two groups was analyzed using Student’s *t*-test for statistical significance. A probability value of <0.05 was considered significant. * *p* < 0.05, ** *p* < 0.01, *** *p* < 0.001, and **** *p* < 0.0001.

## 3. Results

### 3.1. Histology Revealed Scar Tissue Formation and Decreased Collagen After Healing

**Figure 1:**
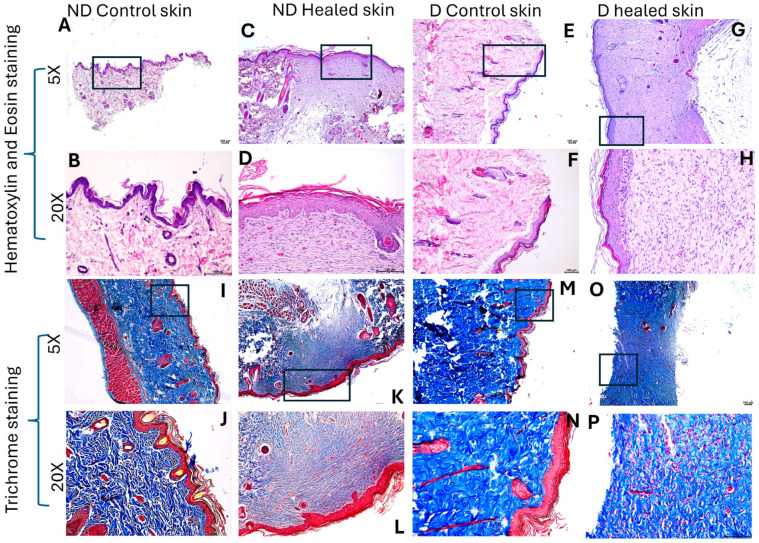
H&E and Trichome staining in nondiabetic (ND) and diabetic (D) control and healed tissues. Panels (**A**–**H**) (H & E staining) and panels (**I**–**P**) (trichrome staining). Panels (**A**–**D**) and (**I**–**L**) are control tissues (nondiabetic), while panels (**E**–**H**) and (**M**–**P**) are tissues from diabetic rats. The square in 5× panels represents the area scanned at 20×.

H&E staining (Figure 1A–H) revealed scar tissue formation in the healed tissues in both control and diabetic rats. The healed tissues were devoid of hair cells, sweat glands, and sebaceous glands. The healed as well as control tissues did not show any inflammation. The healed tissues showed a deranged architectural arrangement with decreased cellularity in diabetic healed tissues. Trichrome staining (Figure 1I–P) showed a decreased amount of collagen (blue staining) in diabetic healed tissues compared to diabetic control and nondiabetic control, and ND healed tissues. On a scale of 0–5, with 0 as no color and 5 as deep blue color, diabetic healed skin showed 2–3, control skin and diabetic control skin showed 4–5, and control healed skin showed 3–4, suggesting decreased collagens in diabetic healed skin

### 3.2. RT-qPCR

**Figure 2:**
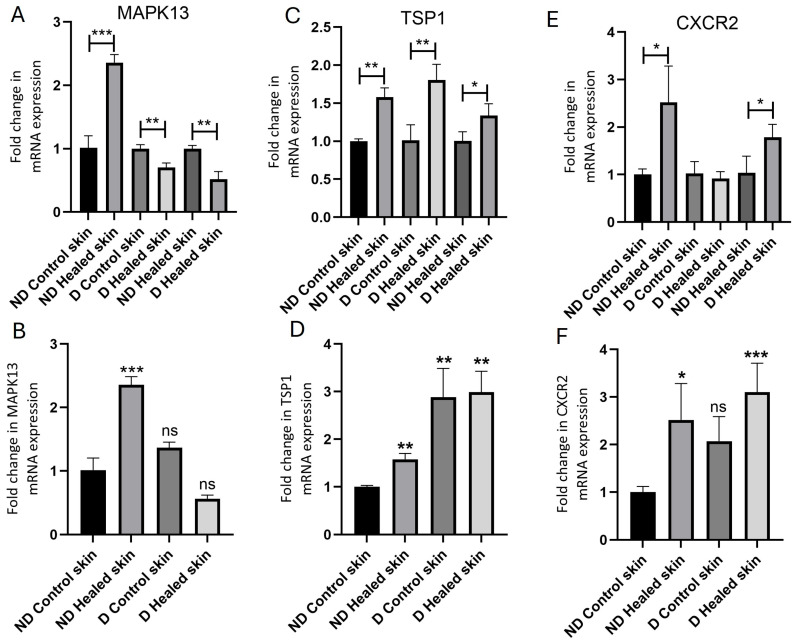
Real-time quantitative polymerase chain reaction (RT-qPCR) analysis of mRNA expression levels of MAPK13 (**A**,**B**), TSP1 (**C**,**D**), and CXCR2 (**E**,**F**). Data are presented as mean ± SD. * *p* < 0.05, ** *p* < 0.01, and *** *p* < 0.001. MAPK13-mitogen-activated protein kinase 13, TSP1-thrombospondin 1, and CXCR2-C-X-C motif chemokine receptor 2. In panels (**A**,**C**,**E**), the gene transcript expression comparison was performed within the group (control with control healed in nondiabetic rats, diabetic controls with diabetic healed rats, and control healed with diabetic healed rats), while in panels (**B**,**D**,**F**), gene transcript expression in all tissues have been compared to control skin from nondiabetic rats. Ns = not significant.

Fold change in mRNA expression was compared between nondiabetic rat skin control and healed, diabetic rat skin control and healed, and nondiabetic healed with diabetic healed. In ND control rats, RT-qPCR analysis showed significantly increased expression of CXCR2 (*p* = 0.027503, Figure 2E), MAPK13 (*p* = 0.000534, Figure 2A), and TSP-1 (*p* = 0.00139, Figure 2C) in healed tissues compared to control skin. Diabetic rats also showed significantly increased TSP-1 expression (*p* = 0.009306) in healed tissue compared to control skin. However, there was a decrease in MAPK13 expression (*p* = 0.005552) in diabetic rats in healed tissue compared to control skin. CXCR2 expression showed no significant increase in the healed compared to control tissues of diabetic rats (*p* = 0.564992). Comparison of healed tissues in control (nondiabetic) versus diabetic rats revealed a significantly increased CXCR2 (*p* = 0.043654, Figure 2E), TSP-1 (*p* = 0.041223, Figure 2C), and MAPK13 (*p* = 0.003105, Figure 2A) expression. Comparison of tissues with control (nondiabetic) skin showed a significantly increased MAPK13 expression (*p* = 0.00053, Figure 2B) in control healed skin, significantly increased TSP1 expression in control healed and diabetic control and healed (*p* = 0.0013, 0.0058, and 0.0014, Figure 2D), and significantly increased CXCR2 expression (*p* = 0.0275 and 0.00042, Figure 2F) in control and diabetic healed skin.

### 3.3. Hyperglycemia Increases the Expression of MAPK13, TSP1, and CXCR2

**Figure 3:**
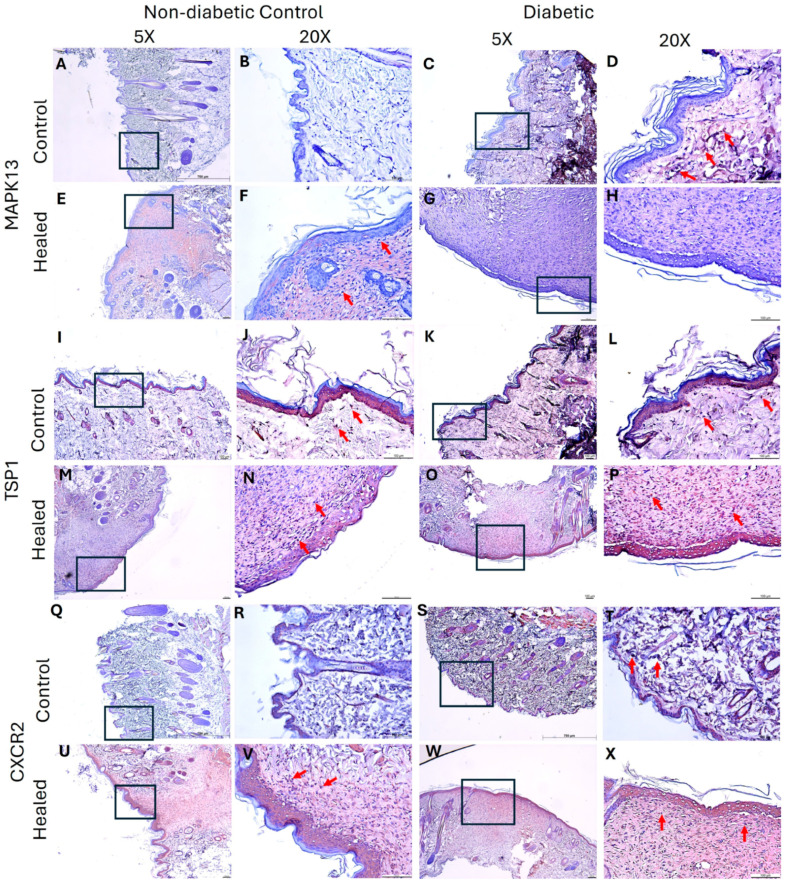
Immunohistochemistry staining of control (skin collected during wounding) and healed (skin collected after wound healed) skin in the control (nondiabetic rats) and diabetic rats. Panels (**A**,**B**,**E**,**F**,**I**,**J**,**M**,**N**,**Q**,**R**,**U**,**V**) (nondiabetic control rats) and panels (**C**,**D**,**G**,**H**,**K**,**L**,**O**,**P**,**S**,**T**,**W**,**X**) (diabetic rats). MAPK13 (panels (**A**–**H**)), TSP1 (panels (**I**–**P**)), and CXCR2 (panels (**Q**–**X**)). Control skin collected during wounding in control and diabetic rats (panels (**A**–**D**,**I**–**L**,**Q**–**T**)) and skin collected during sacrifice after the wound healed (panels (**E**–**H**,**M**–**P**,**U**–**X**)). The squares/rectangles in 5× show the area scanned at 20×. MAPK13-mitogen-activated protein kinase 13, TSP1- TSP1-thrombospondin 1, and CXCR2-C-X-C motif chemokine receptor 2. Arrows show the immunopositivity for the respective protein.

Immunohistochemistry staining revealed increased immunopositivity for MAPK13 in the control skin of diabetic rats (Figure 3D). MAPK13 immunopositivity increased in control and diabetic healed tissues (Figure 3F,H), and control diabetic (Figure 3D) compared to nondiabetic control (Figure 3B). MAPK13 expression was diffuse in healed tissues but was localized in control tissues. Like MAPK13, the immunopositivity of TSP1 was increased in healed tissues, but diabetic healed tissues revealed more immunopositivity than control healed tissues (Figure 3N,P). The immunopositivity was both diffuse and localized in healed tissues. In the control skin, the immunopositivity was localized and seemed comparable between the ND control and diabetic tissues (Figure 3I–L). CXCR2 immunopositivity increased in control diabetic tissues (Figure 3T) compared to ND control (Figure 3R). CXCR2 immunopositivity increased in both ND control and diabetic healed tissues (Figure 3U–X) was more than the respective ND control and diabetic control tissues (Figure 3Q–T). Between the control and diabetic healed tissues, immunopositivity was more in diabetic healed tissues (Figure 3U,W).

### 3.4. Hyperglycemia Regulates Protein Expression of MAPK13, TSP1, and CXCR2

**Figure 4:**
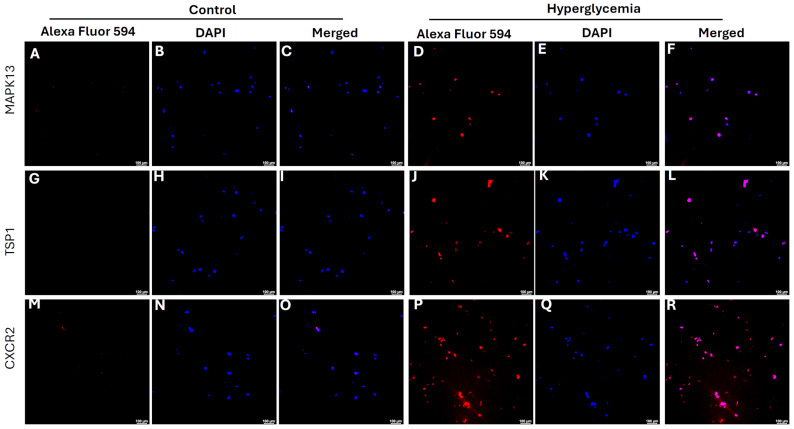
Immunofluorescence staining for the expression of MAPK13 (mitogen-activated protein kinase 13), TSP1 (thrombospondin 1), and CXCR2 (C-X-C motif chemokine receptor 2) in rat fibroblasts. All images were scanned at 100 µm. Panels (**A**,**D**,**G**,**J**,**M**,**P**) are AlexaFluor 594 (red) for protein, panels (**B**,**E**,**H**,**K**,**N**,**Q**) are DAPI-stained nucleus, and panels (**C**,**F**,**I**,**L**,**O**,**R**) are merged images.

In vitro studies showed that hyperglycemia increases the protein expression of MAPK13 (Figure 4D), TSP1 (Figure 4J), and CXCR2 (Figure 4P) at 24 h in rat fibroblasts compared to control cells, as evidenced by increased fluorescence.

### 3.5. Hyperglycemia Significantly Increases Gene Expression of MAPK13, TSP1, and CXCR2

**Figure 5:**
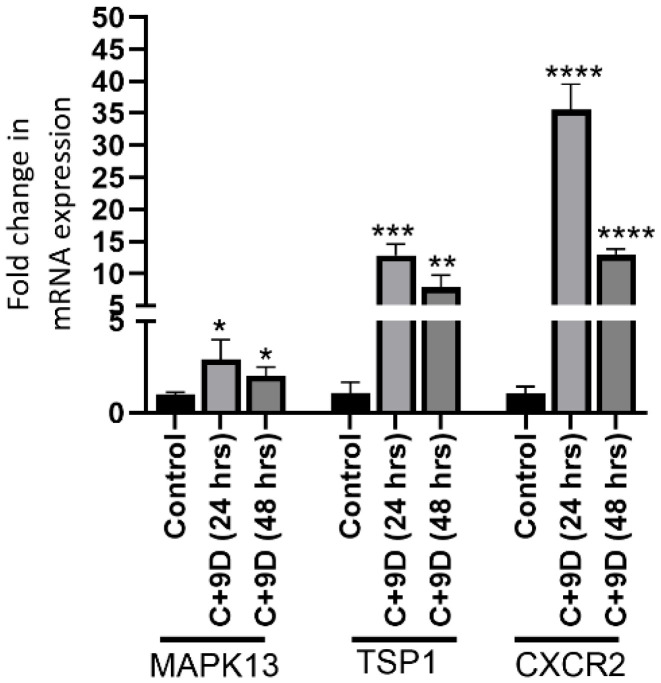
Real-Time quantitative polymerase chain reaction (RT-qPCR) for mRNA expression of MAPK13 (mitogen-activated protein kinase 13), TSP1 (thrombospondin 1), and CXCR2 (C-X-C motif chemokine receptor 2) in rat fibroblasts at 24 and 48 h. * *p* < 0.05, ** *p* < 0.01, *** *p* < 0.001, and **** *p* < 0.0001.

In vitro studies showed significantly increased mRNA expression of MAPK13 (*p* = 0.0384 and 0.0215), TSP1 (*p* = 0.0003 and 0.0037), and CXCR2 (*p* = 0.00011 and 0.00003) at 24 h and 48 h (Figure 5).

## 4. Discussion

Histological results revealed healed tissues with scar formation in both control and diabetic rats without any focus of inflammation. Trichrome staining revealed decreased collagen (blue color intensity) in the healed tissues compared to their respective control, and collagen content was decreased in diabetic tissues compared to control nondiabetic tissues. A decrease in collagen content may be due to impaired wound healing in the presence of hyperglycemia. Hyperglycemia impairs wound healing by damaging dermal fibroblasts and decreasing collagen content and quality. This occurs through mechanisms such as glycation, which weakens collagen structure, and increased oxidative stress, which disrupts cell function and migration. The result is a prolonged inflammatory phase, inadequate tissue regeneration, and poor structural integrity of the healed wound [40,41]. Scar formation in control rats is due to the normal process of wound healing (the wound healed in 14 days), while in diabetic rats, scar formation is due to increasing oxidative stress, disrupting normal cellular processes (wound healed in 21 days) [42]. Scar formation at both time points suggests that remodeling has not happened and may take a longer time. This aspect was not evaluated in this study because the aim of the original ongoing study is to evaluate the healing time only.

Gene transcript expression analysis revealed increased MAPK13 expression in control rats’ healed tissues compared to control skin, and this may be due to the role of MAPK13 in wound healing, as it promotes wound healing, particularly epithelial repair, by coordinating cell growth, migration, and differentiation. MAPK13 activation is necessary for coordinated cell movement after epithelial damage, with its signaling pathway playing a crucial role in the re-epithelialization and structural remodeling required for healing [43,44]. Normally, hyperglycemia increases the expression of MAPK13 [45], and this notion is supported by our immunofluorescence and in vitro PCR findings of increased MAPK13 in fibroblasts expressing p38δ with hyperglycemia. Increase in MAPK expression with hyperglycemia leads to activation of p38MAPK pathways involving increased expression of specific MAPK family members in various cell types, including fibroblasts [26,46]. However, the gene expression of MAPK13 decreased in diabetic healed skin compared to the respective control. A decreased expression of MAPK13 in diabetic healed tissues may be due to decreased fibroblast and endothelial cell density in healed tissues of diabetic rats. This notion is supported by the fact that hyperglycemia decreases cellularity in a healing wound by impairing the function and migration of key cells needed for repair, such as fibroblasts and keratinocytes. This occurs due to increased oxidative stress, inflammation, and altered signaling pathways that disrupt cellular processes like migration, proliferation, and adhesion [47]. Fibroblasts (specifically activated myofibroblasts) are lost in the late stages of normal wound healing, primarily through programmed cell death (apoptosis) or senescence, which shifts them from producing extracellular matrix (ECM) to degrading it, thus resolving scarring and allowing tissue remodeling to finish. This controlled removal stops excessive ECM buildup, controlling fibrosis and promoting scar maturation, unlike in chronic conditions where they persist [48,49]. Hyperglycemia also attenuates angiogenesis by decreasing endothelial cell proliferation, migration, and survival [50]. This may also contribute to decreased MAPK13 in diabetic skin, and this is supported by the results of decreased MAPK13 in diabetic control and healed skin (Figure 2B). A decrease in protein expression of MAPK13 in diabetic healed wounds and an increase in control healed wounds is further supported by the findings of decreased immunohistochemistry (Figure 3F,M). A decrease in MAPK13 in diabetic tissues at day 21 may be explained by the fact that a decrease in MAPK13 activity, particularly after the initial acute phase, is beneficial for normal, scar-free tissue remodeling. Inhibiting MAPK13 has been shown in mouse models and human cell studies to down-regulate basal-ESC reprogramming and prevent the structural remodeling associated with chronic post-injury disease [44].

Next, the results revealed an increased gene and protein expression of TSP1 in diabetic healed skin tissues compared to control nondiabetic skin. TSP1 is an anti-angiogenic mediator that suppresses neo-angiogenesis required during wound healing. This increase is triggered by high-glucose environments and involves complex molecular pathways, often leading to the activation of transcription factors that bind to the TSP-1 promoter [51]. Increase in TSP1 protein expression was more in diabetic healed tissues (Figure 3P) compared to control healed tissues (Figure 3N). To our surprise, TSP1 expression was increased in control healed tissues, which is against the hypothesis because angiogenesis promotes wound healing, and increased TSP1 cannot increase angiogenesis. But it should be noted that the increase in TSP1 expression in the control-healed was less than in diabetic tissues. This increase in physiological wound healing in control tissues may be due to a compensatory mechanism that promotes repair and regulates inflammation. This occurs through its roles in promoting cell migration and proliferation, activating growth factors like TGF-β1, modulating the extracellular matrix, and controlling the inflammatory response by enhancing phagocytosis of damaged cells and regulating angiogenesis [52,53]. Increase in TSP1 in both control and diabetic tissues may also be due to increased platelets after wounding, increased adiposity in diabetics, and an increase in endothelial cells, fibroblasts, vascular smooth muscle cells, and epithelial cells during wound healing [54]. Increase in TSP1 expression with hyperglycemia is supported by the findings of immunofluorescence and in vitro studies, revealing increased mRNA transcript at 24 and 48 h. Increase in TSP1 expression during wound healing in control tissues may also be supported by the fact that TSP1 has beneficial roles in early healing. TSP1 activates TGF-β1 (a key growth factor involved in cell proliferation, extracellular matrix (ECM) deposition (like collagen), and immune response, essential for rebuilding tissue) and regulating cell behavior (migration, adhesion, angiogenesis). TSP1 helps coordinate epithelial cell migration, strengthens cell–matrix adhesion, and remodels the actin cytoskeleton, allowing cells to move into the wound area effectively. While often known as an anti-angiogenic factor, TSP1 also helps manage blood vessel formation in early phase of healing and can promote vessel normalization during later stages of closure [52,55,56].

CXCR2 is a receptor for interleukin (IL)-8 cytokine signaling, which plays a critical role in wound healing. Initially, IL-8 promotes acute inflammation to clear wound debris and promote angiogenesis; however, persistently increased level of IL-8 contributes to chronic inflammation and attenuate angiogenesis [8]. Increased expression of CXCL8 (IL-8) has been associated with chronic nonhealing diabetic ulcers [57]. It should also be noted that high blood glucose levels lead to higher IL-8 production in various cells, including endothelial cells, keratinocytes, and gingival epithelial cells, through mechanisms like enhanced oxidative stress and activation of signaling pathways such as AP-1 and p38. This increase in IL-8 is associated with poor metabolic control in conditions like diabetes and can contribute to inflammation and tissue damage [58,59]. Our findings of increased CXCR2 in diabetic control and healed tissues, as well as in fibroblasts (in vitro studies), further support the notion that hyperglycemia increases expression of CXCR2 and increased IL-8 signaling may contribute to delayed wound healing.

Overall, the results of this study suggest that hyperglycemia modulates the expression of MAPK13, TSP1, and CXCR2. Since these mediators play a critical role in wound healing, they may be an attractive target in diabetic foot ulcers to promote healing by increasing MAPK13 and decreasing TSP1 and CXCR2 in chronic nonhealing wounds. Targeting MAPK13 to promote wound healing and its translational aspect is supported by the finding of Wu et al. [44] reporting MAPK13 mediated control for structural remodeling and disease after epithelial injury in C57BL/6J mice and decreased basal epithelial stem cell growth in human model after *MAPK13* gene knockdown. Other studies using cell lines [43] and C3H mice [60] suggest that MAPK/ERK pathway generally promotes wound healing by increasing VEGF, promoting angiogenesis and boosting keratinocyte proliferation, and enhancing extracellular matrix remodeling via MMPs, thus accelerating the proliferative phase. MAPK13 was activated by selenomethionine [43] and promoted using BRAF inhibitors [60]. These findings further support the translational aspect of targeting MAPK13 to promote healing. TSP1 has an anti-angiogenic effect but studies suggest that attenuating TSP1 can promote wound healing by preventing excessive fibrosis and scarring, though TSP1 also has beneficial roles in early healing. Research using in vitro cells [61] and mice model focuses on blocking its pro-fibrotic actions (like activating TGF-β1 and impairing fibroblast migration) using techniques like antisense oligomers or targeting its receptors (CD47, CD36) to improve skin and corneal repair, showing promise in slowing excessive scarring while allowing normal tissue regeneration [61,62]. These results support the notion of targeting TSP1 to promote healing in chronic wounds.

## 5. Conclusions

The results of this study indicate the effects of hyperglycemia on the gene and protein expression of MAPK13, TSP1, and CXCR2, the mediators with a critical role in wound healing. Diabetic foot ulcers are chronic nonhealing ulcers where decreased angiogenesis, chronic inflammation, and altered extracellular matrix remodeling play a crucial role in pathophysiology. MAPK13 is involved in cell proliferation, migration, and survival; TSP1 is an anti-angiogenic factor; and increased CXCR2 will promote inflammation. Thus, targeting these mediators to promote wound healing in DFUs may have therapeutic significance.

Study limitations and future direction: This study highlights the role of hyperglycemia in modulating the gene and protein expression of MAPK13, TSP1, and CXCR2. Despite these insightful results, there are some limitations. First, the tissues should be evaluated for angiogenesis, ECM components, and inflammation. Since these tissues were used from another ongoing study, we have evaluated these aspects and are preparing a manuscript to submit. The original project was in collaboration with a company, and as per the contract, we will publish the remaining results with their consent. Next, we used fibroblasts for in vitro studies; the effects of hyperglycemia on these mediators should be investigated using other cell types, including endothelial cells, vascular smooth muscle cells, and immune cells, mainly macrophages. Further, we are not reporting the wound healing results, as these results, along with the original research, have been submitted, and the manuscript is under review. Furthermore, the role of these mediators on angiogenesis, ECM components expression, and inflammation in the presence of hyperglycemia may be investigated by using knockdown strategies or increasing their expression. Lastly, the effects of inflammation on the expression of MAPK13, TSP1, and CXCR2 by increasing IL-8 concentrations using in vitro studies should be evaluated. This will support the notion that diabetes-mediated inflammation plays a role in modulating the expression of these mediators and contributes to delayed healing.

## Figures and Tables

**Table 1:** Forward and reverse primer sequences for RT-qPCR.

	Forward Primer (5′—3′)	Reverse Primer (5′—3′)
MAPK13	GAATGACTAGGGTCTGCTTCTG	GCCTGGGCTACATGTGAATA
TSP-1	CCTCGTCACATTGGCTGGAA	CTTGAGTCTGGCCTACGGTTTT
CXCR2	CTGCTGGCTTCCCTACAACA	ATCTCGTTCTGGCGTTCACA
18S	GTAACCCGTTGAACCCCATT	CCATCCAATCGGTAGTAGCG

## Data Availability

All data included in the manuscript.

## Abbreviations

The following abbreviations are used in this manuscript:

AP-1	Activating protein 1
CXCR2	C-X-C chemokine receptor 2
DFUs	Diabetic foot ulcers
DAPI	4′,6-diamidino-2-phenylindole
ECM	Extracellular matrix
ERKs	Extracellular signal-regulated kinases
IL	Interleukin
JNKs	c-Jun N-terminal kinases
MAPK	Mitogen-activated protein kinase
PBS	Phosphate-buffered saline
PCR	Polymerase chain reaction
TSP1	Thrombospondin 1
TGF-β1	Transforming growth factor beta 1
